# A genetic basis for the variation in the vulnerability of cancer to DNA damage

**DOI:** 10.1038/ncomms11428

**Published:** 2016-04-25

**Authors:** Brian D. Yard, Drew J. Adams, Eui Kyu Chie, Pablo Tamayo, Jessica S. Battaglia, Priyanka Gopal, Kevin Rogacki, Bradley E. Pearson, James Phillips, Daniel P. Raymond, Nathan A. Pennell, Francisco Almeida, Jaime H. Cheah, Paul A. Clemons, Alykhan Shamji, Craig D. Peacock, Stuart L. Schreiber, Peter S. Hammerman, Mohamed E. Abazeed

**Affiliations:** 1 Department of Translational Hematology Oncology Research, Cleveland Clinic, 9500 Euclid Avenue/R40, Cleveland, Ohio 44195, USA; 2 Department of Genetics, Case Western Reserve University, 2109 Adelbert Road/BRB, Cleveland, Ohio 44106, USA; 3 Department of Radiation Oncology, Seoul National University College of Medicine, 101, Daehak-Ro, Jongno-Gu, Seoul 110-774, Korea; 4 Broad Institute of MIT and Harvard, 415 Main Street, Cambridge, Massachusetts 02142, USA; 5 Department of Medical Oncology, Dana-Farber Cancer Institute, 450 Brookline Avenue, Boston, Massachusetts 02215, USA; 6 Department of Thoracic and Cardiovascular Surgery, Cleveland Clinic, 9500 Euclid Avenue/J4-1, Cleveland, Ohio 44195, USA; 7 Department of Hematology and Medical Oncology, Cleveland Clinic, 9500 Euclid Avenue/R40, Cleveland, Ohio 44195, USA; 8 Department of Pulmonary Medicine, Cleveland Clinic, 9500 Euclid Avenue/M2-141, Cleveland, Ohio 44195, USA; 9 Center for the Science of Therapeutics, Broad Institute, 415 Main Street, Cambridge, Massachusetts 02142, USA; 10 Department of Chemistry and Chemical Biology, Harvard University, 12 Oxford Street, Cambridge, Massachusetts 02138, USA; 11 Howard Hughes Medical Institute, Broad Institute, Cambridge, Massachusetts 02142, USA; 12 Department of Radiation Oncology, Cleveland Clinic, 9500 Euclid Avenue/T2, Cleveland, Ohio 44195, USA

## Abstract

Radiotherapy is not currently informed by the genetic composition of an individual patient's tumour. To identify genetic features regulating survival after DNA damage, here we conduct large-scale profiling of cellular survival after exposure to radiation in a diverse collection of 533 genetically annotated human tumour cell lines. We show that sensitivity to radiation is characterized by significant variation across and within lineages. We combine results from our platform with genomic features to identify parameters that predict radiation sensitivity. We identify somatic copy number alterations, gene mutations and the basal expression of individual genes and gene sets that correlate with the radiation survival, revealing new insights into the genetic basis of tumour cellular response to DNA damage. These results demonstrate the diversity of tumour cellular response to ionizing radiation and establish multiple lines of evidence that new genetic features regulating cellular response after DNA damage can be identified.

## Abstract (web summary)

The variability in patient response to radiation treatment is difficult to predict. Here, using more than 500 cell lines the authors measure response to radiation exposure and a large panel of compounds, and show that response can be predicted by genetic alterations of the cells.

## Graphical abstract

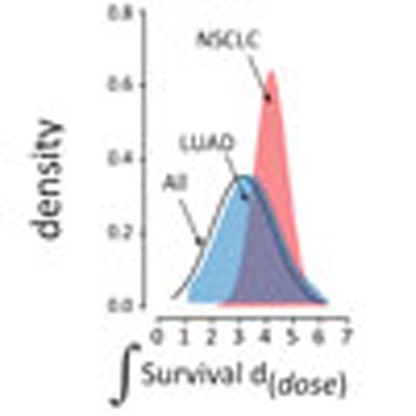

Clinical radiotherapy has made significant advances since its inception, growing into a tertiary specialty with significant contributions to curative and palliative treatments of cancer and healthcare cost^1^. A major limitation to its appropriate application, however, has been the lack of measurable biological indicators, or biomarkers, that can reliably identify patients with cancers that are more or less likely to respond to these treatments^2^,^3^.

Advances in genomic technology have enabled a cataloguing of cancer genes that has resulted in the identification of genetic alterations that contribute to oncogenesis and/or tumour progression and in some cases has led to significant therapeutic advances^4^,^5^,^6^,^7^. In contrast, X-rays and DNA-damaging drugs are delivered based on the site of anatomical origin of the disease and do not currently take into account the genetic complexity that may regulate therapeutic response. Herein, using data derived from a single experimental platform and analysed using a rigorous statistical methodology, we study the genetic determinants of survival after radiation in 533 human cancer cell lines across 26 cancer types. These results reveal new insights into the intrinsic determinants of tumour cellular response to DNA damage.

## Results

### Variation in survival after irradiation

**Figure 1:**
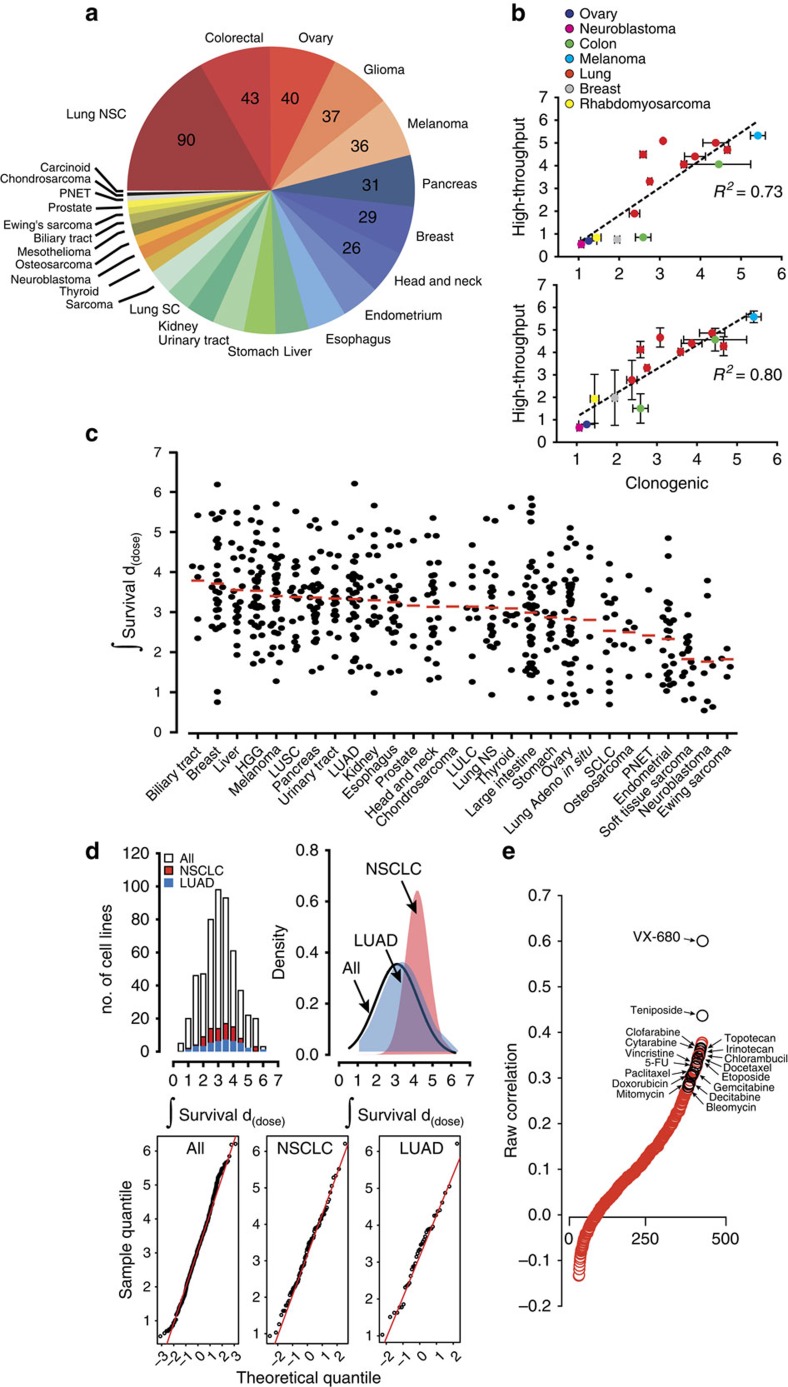
Variation in cancer cell line survival after radiation-induced damage. (**a**) Distribution of cancer types profiled by lineage. (**b**) The high-throughput platform accurately profiles cancer cell lines. Integral survival was calculated for each cell line profiled by the high-throughput platform (*n*=1 (top), *n*=2 (bottom)) and by clonogenic survival measurements (*n*≥2). Scatter plots, linear regression, and *R*^2^ values were calculated comparing the integral survivals of the high-throughput platform to clonogenic survival. Data are expressed as the means±s.e.m. (**c**) Integral survival is displayed by column scatter plot separated by lineage and histology where appropriate. (**d**) Histogram, probability density function, and Normal Q–Q plots analyses of calculated integral survival of 533 cell lines (*‘All'*), 89 non-small cell lung cancer cell lines (*‘NSCLC'*), and 39 lung adenocarcinoma cell lines (*‘LUAD'*). (**e**) Correlation of response between radiation and compounds. Spearman correlation coefficient was calculated between integral survival values after exposure to radiation or 481 compounds. Correlation was then plotted relative to correlation rank. Some chemotherapeutic agents in clinical use are shown. HGG, high-grade glioma; LULC, lung large cell; LUSC, lung squamous cancer; PNET, primitive neuroectodermal tumours; SCLC, small cell lung cancer.

We profiled radiation survival of 533 cancer cell lines comprising 26 cancer types using a recently developed high-throughput profiling platform (Fig. 1a)^8^. This platform was previously benchmarked against the clonogenic survival assay in lung squamous cancer cell lines. We previously demonstrated that the high-throughput measurements closely approximated clonogenic survival by most radiation response parameters, with the greatest level of correlation observed with a longer time to readout, at doses within the growth inhibition of 50% (GI_50_) range of most cell lines profiled, and when comparing mean integral survival values. To assess the platform's validity beyond the lung squamous lineage, we measured clonogenic survival in cell lines derived from multiple lineage and exhibiting a wide range of responses to radiation. We integrated survival as a function of dose and generated values for each cell line (Supplementary Fig. 1a and Supplementary Data 1). Integral survival (single experiment) or mean integral survival values (average of duplicates) for 15 cell lines were calculated and compared with values from the clonogenic assay (for each cell line, *n*≥2; Fig. 1b). High-throughput and colony integral survival values were significantly correlated, with Pearson's *r*=0.85, *R*^*2*^=0.73 for single experimental profiling and Pearson's *r*=0.89, *R*^*2*^=0.80 with the average of two profiling experiments. Similar to our previous results, we showed that the proliferation index approximated clonogenic survival across individual doses, with the best approximations occurring within the GI_50_ range (Supplementary Fig. 1b).

A column scatter plot of integral survival demonstrated significant variation in survival across and within lineages (Fig. 1c), the latter being on the order of 5- to 7-fold. To assess differences in the distribution of response across all profiled cancer types, in a category of cancers derived from single organ, and in a single lineage, we plotted the histogram and probability density distribution of integral survival for all profiled cell lines, those derived from non-small cell lung cancers, and those from lung adenocarcinoma (Fig. 1d). All three demonstrated a normal distribution. In fact, the majority of lineages (cut-off ≥25 cell lines profiled), including lung adenocarcinoma, breast, glioma, ovary, pancreas and melanoma, had a response that was normally distributed (D'Agostino-Pearson: *K*^2^<0.65, *P*>0.5). Only two lineages, colorectal and uterine, demonstrated non-Gaussian distributions in response to radiation, mostly due to a higher proportion of resistant cell lines than predicted by a normal distribution (Supplementary Fig. 2). This is attributed to cell lines with large values of copy number. Taken together, the high-throughput platform accurately profiles cell lines from multiple lineages for radiation response and reveals a mostly Gaussian distribution of radiation response within lineages.

### X-rays and DNA-damaging drugs

To assess the accuracy of the platform and probe similarities between radiation and drug therapy, we calculated the correlation of responses between radiation and 481 compound probes profiled by US National Cancer Institute's Cancer Target Discovery and Development (CTD^2^; https://ctd2.nci.nih.gov/dataPortal/ and ref. 9) (Fig. 1e and Supplementary Data 2). The most positively correlated compound, VX-680, is a pan-inhibitor of the Aurora kinases (Spearman's *r*=0.60)^10^. Aurora kinases are essential for the regulation of chromosome segregation and cytokinesis during mitosis and their inhibition leads to cell death by mitotic catastrophe, a major mode of cell death after irradiation. In addition to VX-680, genotoxic chemotherapeutics such as etoposide, paclitaxel, doxorubicin, bleomycin and others were likely to be correlated with radiation sensitivity. These correlations suggest similar genetic dependencies between genotoxic compounds and radiation.

### SCNA regulate survival after irradiation

**Figure 2:**
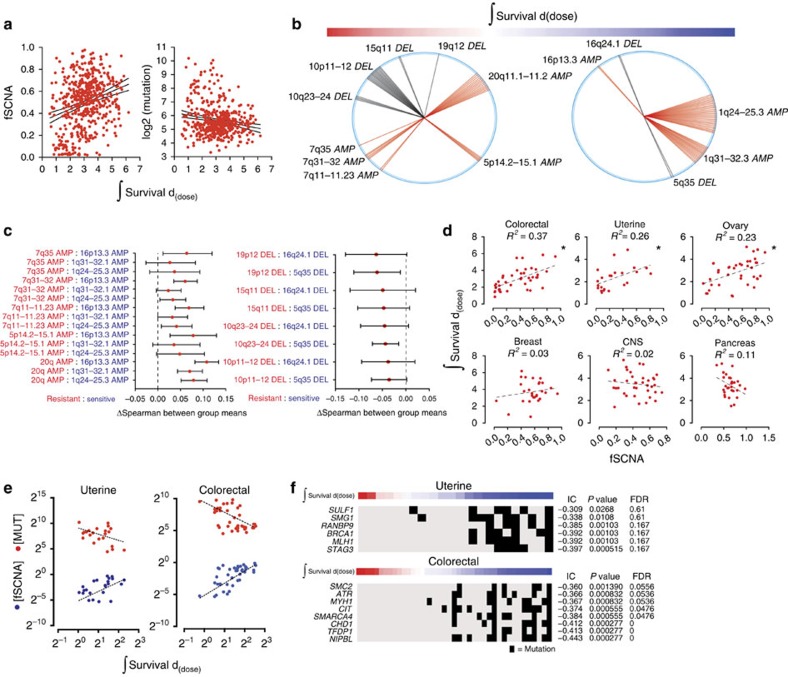
SCNA changes are associated with survival after radiation-induced damage. (**a**) Plots of *f*SCNA, integral survival, and number of mutations per sample. (**b**) The top 50 probes that correlate with radiation resistance (left) and sensitivity (right) are shown. Radii (single probe) or sectors (multiple probes) correspond to chromosome positions. Each radius represents a distinct probe that mapped to the designated chromosome position. (**c**) Individual SCNA can regulate the response to radiation directly. We correlated radiation survival with the expression of genes within the altered segments and compared the means of the coefficients by pairwise analysis, resistant (red) versus sensitive (blue). Spearman means for alterations depicted in (**b**) were analysed by analysis of variance and Tukey Contrasts. 95% confidence level intervals for each pairwise comparison are shown. (**d**) Scatter plots, linear regression, and *R*^*2*^ values of the integral survival and *f*SCNA by lineage. (**e**) Scatter plot and linear regression of integral survival, *f*SCNA, and the number of mutations (MUT) in uterine and colorectal carcinoma. (**f**) Heatmap of integral survival (red=resistant, blue=sensitive) and gene mutations in uterine and colorectal carcinoma cells. Black bar represents a mutation in the corresponding gene.

Somatic copy number alteration(s) (SCNA) are common in cancer^11^, have a critical role in promoting oncogenesis^12^, and an understanding of their phenotypic effects has led to advances in cancer diagnostics and therapeutics^7^,^13^,^14^. The interaction between the SCNA landscape and the response to radiation remains poorly defined. We measured the fraction of the genome that contains a SCNA or (*f*SCNA) by measuring the length of segments with log_2_ SCNA values larger than 0.2 from the Genomic Identification of Significant Targets in Cancer (GISTIC) output (see Methods section) divided by the length of all segments measured. Therefore, the *f*SCNA represents a surrogate measure of genomic instability based on relative SCNA. We observed a positive correlation (Pearson's *r*=0.27) between *f*SCNA and integral survival (Fig. 2a and Supplementary Data 3). We also plotted the log_2_ of the number of mutations per sample and integral survival and observed a modest negative correlation (Pearson's *r*=−0.19).

We reasoned that the overall positive correlation of *f*SCNA with radiation survival could reflect an increased capability of tumour cells to repair DNA double-strand breaks (DSB) after radiation, utilizing mechanisms that are also used in the creation of SCNA such as non-homologous or micro-homology-mediated end joining or other error-prone repair mechanisms (for example, non-allelic homologous recombination)^15^. Alternatively, individual SCNA could regulate survival after radiation by changing the expression of specific genes within the structurally altered chromosomal segments. The former is predicted to create a stochastic order of individual SCNA correlated with survival, the latter would identify discrete SCNA on both sides of the survival spectrum. To assess the association of individual SCNA with radiation response, we correlated alterations with radiation survival using the information co-efficient (IC) (see Methods section and Supplementary Data 4). The top 50 gene-level SCNA correlating with resistance and sensitivity were organized by chromosome position and the results were depicted using a wheel plot (Fig. 2b). The relative enrichment for discrete chromosomal segments that correlated with resistance or sensitivity suggested that individual SCNA events were not randomly distributed across the radiation response range. To assess whether SCNA can contribute to radiation response directly, we correlated radiation survival with the expression of genes within the altered segments and compared the means of the coefficients by pairwise analysis, resistant (red) versus sensitive (blue; Fig. 2c). The amplified regions that correlated with radiation resistance had a significantly higher mean correlation coefficient than amplifications that correlated with radiation sensitivity. The inverse was observed for deleted regions that correlated with radiation resistance. In some cases, changes in the expression of individual genes have previously been implicated in response to cytotoxic stress. For example, genes overexpressed as a consequence of focal amplicon 20q11.2 include functionally important genes in cell cycle regulation (*E2F1*), chromosome maintenance (*KIF3B*), glutathione synthesis (*GSS*) and apoptosis (*BCL2L1*). All of these genes were positively correlated with radiation resistance (Supplementary Data 4). Collectively, these results indicate that SCNA regulate the response of cells to radiation-induced damage in part through direct changes in gene expression.

The frequency and distribution of SCNA vary across tumour lineages^12^. A scatter plot of *f*SCNA and integral survival revealed differences in the degree and the direction of association across lineages. Colorectal, uterine and ovarian carcinomas showed a positive correlation between survival and *f*SCNA values (Fig. 2d). Colorectal and uterine carcinomas have been previously shown to contain tumours with extensive SCNA and low mutation frequency, and a subset of tumours with low SCNA and high mutation frequency. The latter is attributed to either *MLH1* silencing and/or DNA polymerase ɛ (*POLE*) mutations^16^,^17^. Consistent with these findings, we observed a correlation between radiation survival and SCNA and an anti-correlation between radiation survival and mutation frequency in both lineages (Fig. 2e). We identified mutations in individual genes that correlated with radiation sensitivity in both lineages using the IC (Fig. 2f). Of the top 20 genes that correlated with radiation sensitivity in uterine and colorectal cancers, 6 and 8, respectively, have previously been associated with DNA repair. These results suggest an association between low SCNA, high mutation frequency, DNA repair gene disruption and radiation sensitivity in these lineages.

### Gene mutations regulate cellular survival after irradiation

**Figure 3:**
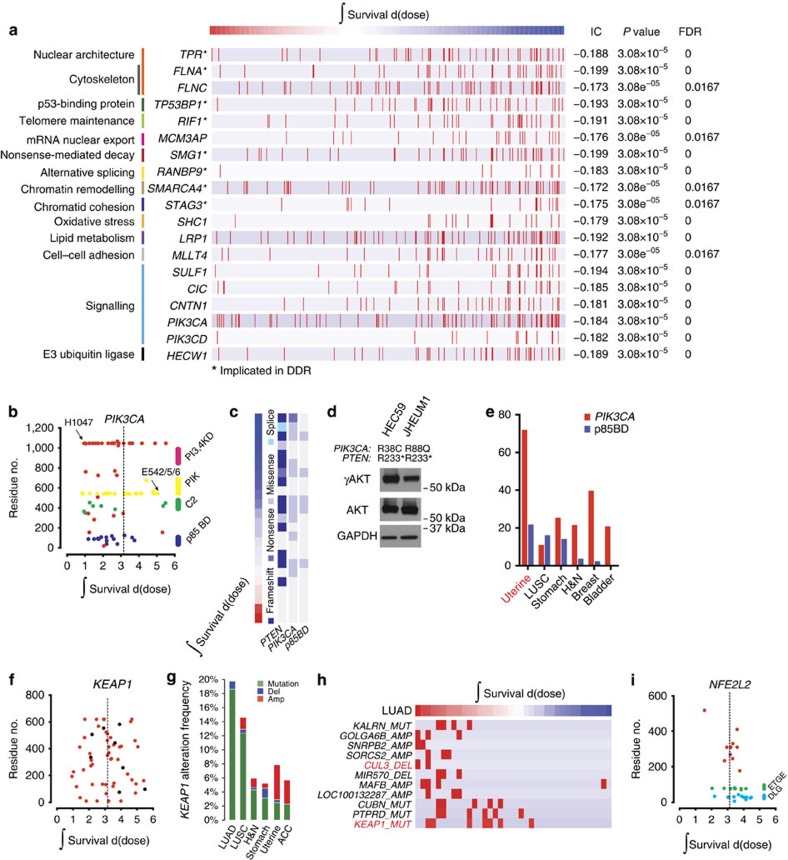
Mutations in genes associated with distinct cellular functions correlate with survival after radiation-induced damage. (**a**) Top 19 genes that when mutated are associated with radiation sensitivity are organized by biological functions. Red bars represent samples with a mutation. (**b**) Scatter plot of integral survival and amino acid position for cell lines containing mutations in *PIK3CA*. (**c**) Association between radiation response and mutation in *PIK3CA* and *PTEN* in uterine carcinoma. (**d**) γAKT, AKT, and GAPDH levels in two uterine cancer cell lines with *p85* BD mutations. (**e**) Frequency of *PIK3CA* and *PIK3CA p85* BD mutations as annotated by TCGA; organized from left to right by frequency of mutations in *p85* BD. (**f**,**i**) Scatter plot of integral survival and amino acid position for cell lines containing mutations in *KEAP1* and *NFE2L2*. (**g**) *KEAP1* alteration frequency by lineage, and sub-lineage where appropriate, as annotated by TCGA. Organized from left to right by frequency of *KEAP1* mutation. (**h**) Association between integral survival and genomic features in lung adenocarcinoma. Red bar represents a copy number change or mutation in the corresponding gene.

Recent studies have identified recurrent gene mutations that are correlated with the likelihood of response to specific agents in cancer^4^,^5^. Identifying gene mutations that correlate with radiation response have the potential to similarly inform clinical management. We identified gene mutations that correlated with radiation sensitivity across all lineages using the IC. We observed higher IC values for genes with mutations and radiation sensitivity compared with resistance (Supplementary Data 5). The top 19 genes that were associated with radiation sensitivity when mutated were organized by biological function (Fig. 3a). Seven of these genes have previously been implicated in the DNA damage response: *TPR*^18^, *FLNA*^19^, *TP53BP1* (ref. 20), *SMG1* (ref. 21), *RANBP9* (ref. 22), *SMARCA4* (ref. 23) and *STAG3* (ref. 24). A subset of the 19 genes demonstrated domain selectivity in conferring sensitivity (Supplementary Fig. 3). Other top genes that correlated with radiation sensitivity have not previously been implicated in radiation-induced damage response.

We sought to determine whether identified genes were regulators of radiation response or merely associated with sensitivity. An example of the latter is *PIK3CA*. The phosphatidylinosoitol 3-kinase (PI3K)/protein kinase B (AKT) pathway is frequently deregulated in human cancer and is an important tumour cell survival pathway^25^. Mutations in *PIK3CA*, which typically result in activation of PI3K/AKT, were associated with radiation sensitivity in our data set (Fig. 3a). However, activation of PI3K/AKT has previously been associated with radiation resistance^26^,^27^. A closer look at the domains within *PIK3CA* and their role in radiation sensitivity indicates that cell lines with mutations in the *p85*-binding domain (*p85* BD) are mostly sensitive to radiation, driving the overall association (Fig. 3b). Cell lines with mutations in the *p85* BD of *PIK3CA* were largely from the uterine lineage (38% of the cell lines profiled in Fig. 3b), had a co-occurring mutation in *PTEN* (Fig. 3c), and two representative cell lines were γAKT replete (Fig. 3d), indicating that *p85* BD mutations retain PI3K enzymatic activity. Mutations in the *PIK3CA p85* BD were also frequently identified (>20%) in uterine tumour samples profiled by The Cancer Genome Atlas (TCGA) network (Fig. 3e), paralleling the cell line data^17^. These results indicate that *PIK3CA p85* BD mutations reflect markers of a radiosensitive lineage (uterine carcinoma).

We analysed mutations that conferred radiation resistance and identified the key regulator of oxidative stress response, *KEAP1*, which ranked ninth (IC=0.112; *P*=0.0513, calculated using the empirical permutation test) from a list of >1,600 genes (Fig. 3f, Supplementary Data 5). We, and others, have shown that mutations in *KEAP1* result in the stabilization and activity of the master transcriptional regulator of oxidative damage response, Nrf2 (encoded by *NFE2L2*), thereby conferring radiation resistance^8^,^28^,^29^. Recently, the spectrum of *KEAP1* mutations was analysed, revealing distinct functional categories including passenger, loss-of-function, hypomorphic or ‘super-binders'^30^. We reasoned that the likelihood of passenger or hypomorphic mutations masking association is less likely to occur in a lineage with frequent *KEAP1* mutations. To test this, we analysed the IC in adenocarcinoma of the lung, which has the highest frequency of *KEAP1* mutations of any lineage profiled by the TCGA network to date (Fig. 3g)^31^. Consistent with TCGA network data, the strongest association between *KEAP1* mutation and radiation resistance was in adenocarcinoma of the lung (IC=0.352; *P*=0.0224, calculated using the empirical permutation test; Fig. 3h). *CUL3*, encoding the ubiquitin ligase adaptor that binds to Keap1 and degrades Nrf2, was also associated with radiation resistance in adenocarcinoma of the lung (Fig. 3h).

To assess the impact of mutation position on the IC, we assessed the relative importance of residue position on survival in the binding partner of Keap1, Nrf2 (Fig. 3i). In human cancer, somatic mutations in *NFE2L2* frequently occur within the two *KEAP1*-binding sites (**D**_**29**_**LG** and **E**_**79**_**TGE**)^32^. The IC value for *NFE2L2* mutation across all lineages (IC=−0.0697; *P*=0.329, calculated using the empirical permutation test) was significantly higher when mutations restricted to the two *KEAP1*-binding sites were considered (IC=0.245; *P*=0.033, calculated using the empirical permutation test).

Taken together, these results describe gene mutation determinants of radiation-induced cellular damage response and reveal distinct functional consequences for categories of mutations within individual genes.

### Gene expression profiles regulate survival after irradiation

We used ssGSEA (single-sample Gene Set Enrichment Analysis) projections^8^,^33^ as a gene set identification tool to find genetic pathways that are differentially correlated with radiation response (see Methods section). We compared the profiles of each gene set/pathway with the radiation response scores (integral survival) across cancer types. The ssGSEA scores are displayed in a heatmap with the top gene sets that correlate and anti-correlate with radiation survival organized by cellular pathways (Supplementary Fig. 4 and Supplementary Data 6). The top gene sets that correlated with radiation sensitivity revealed pathways including DNA damage response, cell cycle, chromatin organization and RNA metabolism. The top gene set that correlated with radiation resistance revealed pathways including cellular signalling, lipid metabolism and transport, stem-cell state, cellular stress and inflammation. The multitude of pathways associated with radiation response indicates that cellular processes well-beyond DNA repair regulate cellular survival after radiation. A closer look at active signalling pathways that correlate with radiation resistance reveals several targetable cellular receptors including transforming growth factor-β, estimated glomerular filteration rate, oestrogen receptor (ER), NFκB, JAK/STAT, AKT, FGFR, HER2, RAF, MEK and Wnt/β-catenin. Some of these receptors have previously been shown to confer resistance to radiation in selected cell lines^34^. These results indicate the broad role these signalling pathways serve in regulating radiation survival across several tumour lineages and implicate new targets for radiosensitization.

**Figure 4:**
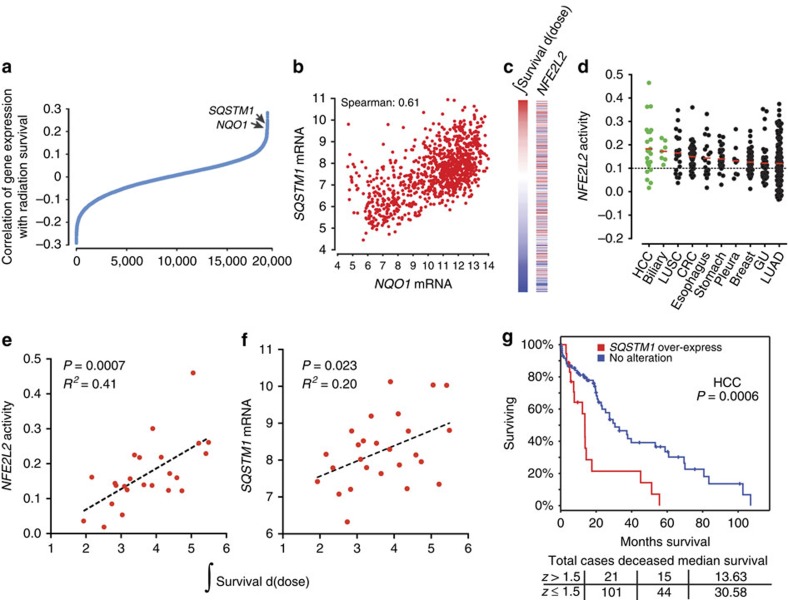
Gene expression changes regulating oxidative stress response are associated with radiation resistance in several cancer lineages. (**a**) Correlation of *NQO1* and *SQSTM1* expression with radiation resistance. Spearman's correlation coefficient was calculated between gene expression and integral survival values. Correlation was then plotted relative to correlation rank. (**b**) Relationship between *NQO1* and *SQSTM1* mRNA expression in CCLE. (**c**) ssGSEA association between *NFE2L2* signature score and integral survival. (**d**) *NFE2L2* is frequently activated in hepatocellular (HCC) and biliary tumours. A column scatter plot of *NFE2L2* signature score for 967 cell lines in the CCLE organized by disease site and histology where appropriate. Solid bars represent the mean in each category. Dashed line represents the median across all CCLE lines. (**e**) *NFE2L2* activity scores and (**f**) *SQSTM1* mRNA levels from HCC and biliary cancer cell lines were plotted as a function of radiation integral survival. (**g**) Kaplan–Meier survival analysis curve calculated from 122 hepatocellular cancer patients from TCGA (https://tcga-data.nci.nih.gov/tcga/); cut-off=*z*>1.5. *z*=+0.8 or greater demonstrated a statistically significant difference in overall survival by the log-rank test.

To assess the importance of the expression of individual genes on radiation survival, we calculated correlation coefficients between 18,988 genes and integral survival values (Fig. 4a; Supplementary Data 7). *NQO1* and *SQSTM1*, both transcriptionally activated by Nrf2 (ref. 35), were the ninth and eleventh genes identified as strongly associated with resistance, corroborating a role for oxidative stress response in radiation resistance already implicated by the gene mutation data. *NQO1* encodes the NAD(P)H-quinone oxidoreductase, an enzyme that detoxifies cells from reactive oxygen species-induced quinone-containing compounds^36^. The ubiquitin binding protein, Sqstm1 (or p62) plays a role in oxidative stress, cellular signalling and autophagy^37^. Sqstm1 has been previously shown to interact with Keap1 and accumulation of Sqstm1 can lead to an increase in Nrf2 activity^38^.

Consistent with these results, *NQO1* and *SQSTM1* gene expressions are strongly correlated across 979 Cancer Cell Line Encyclopedia (CCLE) cell lines (Fig. 4b) and *NFE2L2* transcriptional activity is associated with radiation survival across all lineages (Fig. 4c). *NFE2L2* transcriptional activity plotted by lineage revealed the highest overall activity in hepatocellular carcinoma (HCC) and biliary cancer (Fig. 4d). We have shown that Nrf2 is mainly activated by mutations in *NFE2L2* and/or *KEAP1* and/or deletions in *CUL3* in lung squamous cancers^8^. Similar gene alterations have not been identified in HCC or biliary cancer (TCGA network). Instead, recent reports suggest an important role for *SQSTM1* in Nrf2 activation in HCC^38^. To test the association between Nrf2 and Sqstm1 activity and radiation survival in HCC, we plotted integral survival values with *NFE2L2* activity (Fig. 4e) and *SQSTM1* expression (Fig. 4f). We found that HCC had the strongest association between radiation survival and Nrf2 activity in any lineage profiled (*R*^*2*^=0.41 in HCC versus *R*^*2*^=0.22 in LUSC). *SQSTM1* expression was also correlated with radiation survival, albeit at a lower level than that obtained with the Nrf2 score. We attributed this to noise associated with single-gene expression measurements, compared with a composite Nrf2 score that includes 565 genes. HCC is commonly managed by genotoxic therapies (chemo- and/or radio-embolization, external beam radiotherapy) and/or surgery, suggesting that patients who resist genotoxic stress may have poorer clinical outcomes. HCC patients with elevated *SQSTM1* expression have a significantly lower overall survival, indicating a poor overall prognosis for patients with active Nrf2 in HCC (Fig. 4g; TCGA network). This is analogous to the poor prognosis of NSCLC patients with active Nrf2 (ref. 39 and Supplementary Fig. 5).

### Radiogenomic profiling of breast cancer

For many women with breast cancer, breast-conserving surgery or mastectomy can result in the removal of detectable macroscopic disease. However, tumour foci might remain in local and regional tissue such as the intact breast or chest wall and/or regional lymph nodes. Tumour recurrence can cause considerable morbidity, dissemination of disease and an increased probability of breast cancer mortality^40^,^41^. Radiotherapy significantly decreases the risk of local and regional recurrence and breast cancer mortality^42^,^43^. Despite the demonstrated efficacy of breast radiotherapy, there remains an important need for identifying patients who are more likely to fail therapy and improving radiation treatments in those patients.

**Figure 5:**
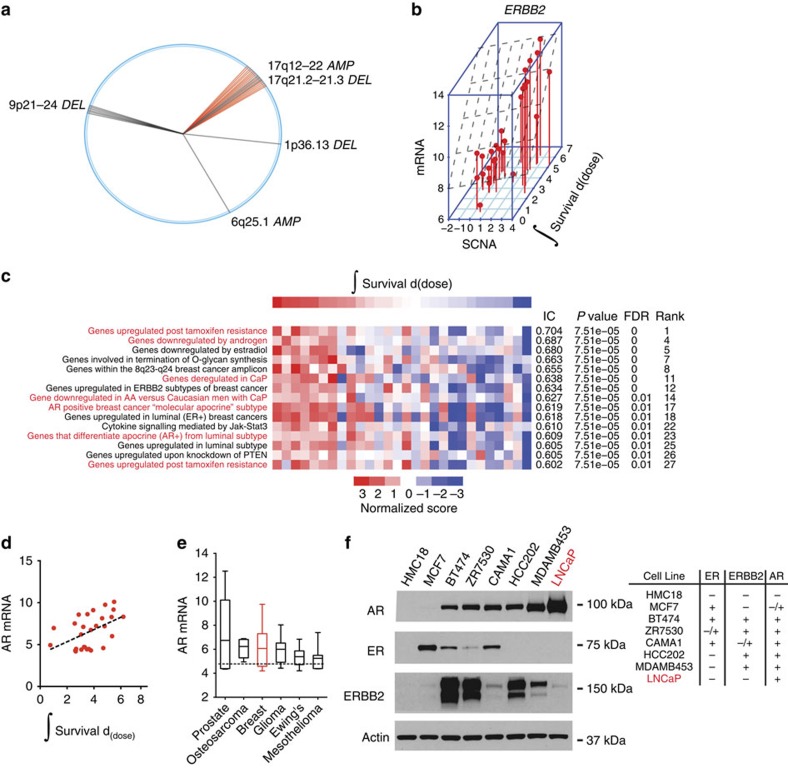
Genes associated with survival after radiation-induced damage in breast carcinoma. (**a**) The top 50 copy number probes associated with radiation resistance in breast adenocarcinoma are shown. Radii (single probe) or sectors (multiple probes) correspond to chromosome positions. (**b**) *ERBB2* amplification is associated with survival after radiation-induced damage. Three-dimensional scatter plot of integral survival, *ERBB2* copy number, and *ERBB2* mRNA expression. (**c**) ssGSEA identifies gene sets that correlate with resistance to radiation. Heatmap of ssGSEA scores (red=positive, blue=negative). A subset of the top 27 gene sets is shown (see Supplementary Data 10 for MSigDB gene set names). Genes sets depicted in red font are associated with androgen signalling. (**d**) Scatter plot and linear regression of *AR* mRNA levels and radiation integral survival in breast cancer. (**e**) *AR* is frequently expressed in multiple cancer lineages. A box and whiskers plot of *AR* expression for 967 cell lines in the CCLE organized by lineage. Whiskers represent minimum and maximum values. (**f**) (Left) Relative AR, ER, and HER2 protein levels in whole-cell extracts from breast cancer cell lines and LNCaP, a prostate cancer cell line. (Right) Annotation of expression based on western analysis include: −(no observable expression),+(expression) or −/+ (faint expression was observed with longer exposure to film, Supplementary Fig. 7).

To identify genetic determinants of radiation survival in breast carcinomas, we began with an unbiased query of gene mutations or copy number changes that correlated with radiation survival in 28 breast cancer cell lines (Supplementary Data 8). The top 50 segments correlating with resistance were organized by chromosomal position and the results were depicted using a wheel plot (Fig. 5a). Amplification of 17q12–22, which contains *ERBB2*, was associated with radiation resistance. Cell lines containing 17q12–22 amplification had elevated *ERBB2* copy number and messenger RNA (mRNA), and were generally derived from tumours clinically annotated as having *ERBB2* amplifications (Supplementary Data 9). *ERBB2* copy number (Pearson's *r*=0.43) and gene expression (Pearson's *r*=0.45) were correlated with radiation survival (Fig. 5b). These results suggest that *ERBB2* is the likely mediator of radiation resistance in the 17q12–22 amplicon. Consistent with these results, overexpression of *ERBB2* has previously been shown to confer therapeutic resistance in breast cancer cells and Trastuzumab, a monoclonal antibody that interferes with ErbB2, sensitizes ErbB2 expressing cells to radiation^44^.

### *AR* regulates survival after irradiation in breast cancer

To find additional genetic pathways that are differentially correlated with radiation response, we applied ssGSEA projections (Fig. 5c). Multiple pathways were correlated with radiation resistance (*ER*, *ERBB2*, JAK/STAT3 and PI3K). However, one of the most correlated and intriguing gene sets identified was associated with androgen signalling. Androgen receptor (AR) expression is most frequently observed in prostate cancer, where it has been shown to promote resistance to radiation^45^. Several clinical studies have demonstrated a benefit in overall survival with the combination of androgen-deprivation therapy and radiation compared with radiation alone in patients with intermediate- and high-risk prostate cancer^46^,^47^,^48^. Although its role in breast oncogenesis remains poorly defined, AR is detected in a majority of breast carcinomas at levels greater than normal breast levels^49^. A subset of AR-positive, triple-negative breast carcinomas, which lack ER and progestrone receptor (PR) expression and ErbB2 overexpression, appear to be dependent on AR signalling for growth^50^,^51^. A phase II study that explored bicalutamide in AR-positive, ER/PR-negative metastatic breast cancer showed a modest clinical benefit^52^. A single-arm phase II is currently assessing the more potent AR antagonist, enzalutamide (ENZ), in women with advanced, AR-positive, ER/PR-negative breast cancer. Therefore, the clinical effectiveness of anti-androgens as single-agent therapy in the management of breast cancer patients remains unknown. Based on our initial results and these observations, we sought to examine the role of AR and test the rationale for combining androgen blockade and radiation in breast cancer, as is the standard of care in locally advanced prostate cancer^46^,^47^.

We showed that AR mRNA levels correlated with radiation survival (Pearson's *r*=0.48; Fig. 5d). We analysed data from the CCLE to determine the relative mRNA levels of AR across lineages. Prostate cancers had the highest mean value among 28 tumour types followed by osteosarcoma and breast cancer (Fig. 5e). We determined an association between *AR* mRNA and protein levels (Pearson's *r*=0.615) and *AR* and *ESR1* (or ERα) mRNA (Pearson's *r*=0.34) in TCGA patient samples (Supplementary Fig. 6). A subset of TCGA samples expressed *AR* and not *ESR1*. Consistent with this data, we showed that some of the breast carcinoma cell lines that we profiled expressed AR with varying levels of ER (and ErbB2; Fig. 5f and Supplementary Fig. 7).

MDAMB453 cells expressed AR but not ER, and although ErbB2 was overexpressed in these cells, the level of expression was significantly lower than that observed in other AR-positive cell lines (BT474, ZR7530 or HCC202; Fig. 5f). To examine the activity of ErbB2 in this cell line, we measured γErbB2 levels at tyrosine 1,248 (ref. 53). γErbB2 was not elevated in MDAMB453 cells, indicating that ErbB2 is not activated in this cell line (Supplementary Fig. 8). These results indicate that *ERBB2* overexpression is not sufficient to activate ErbB2 in MDAMB453 cells. Taken together, MDAMB453 cells are AR-positive, ER-negative and ErbB2 inactive, consistent with its gene expression-based classification into the luminal AR-expressing subtype of triple-negative breast cancer^54^.

**Figure 6:**
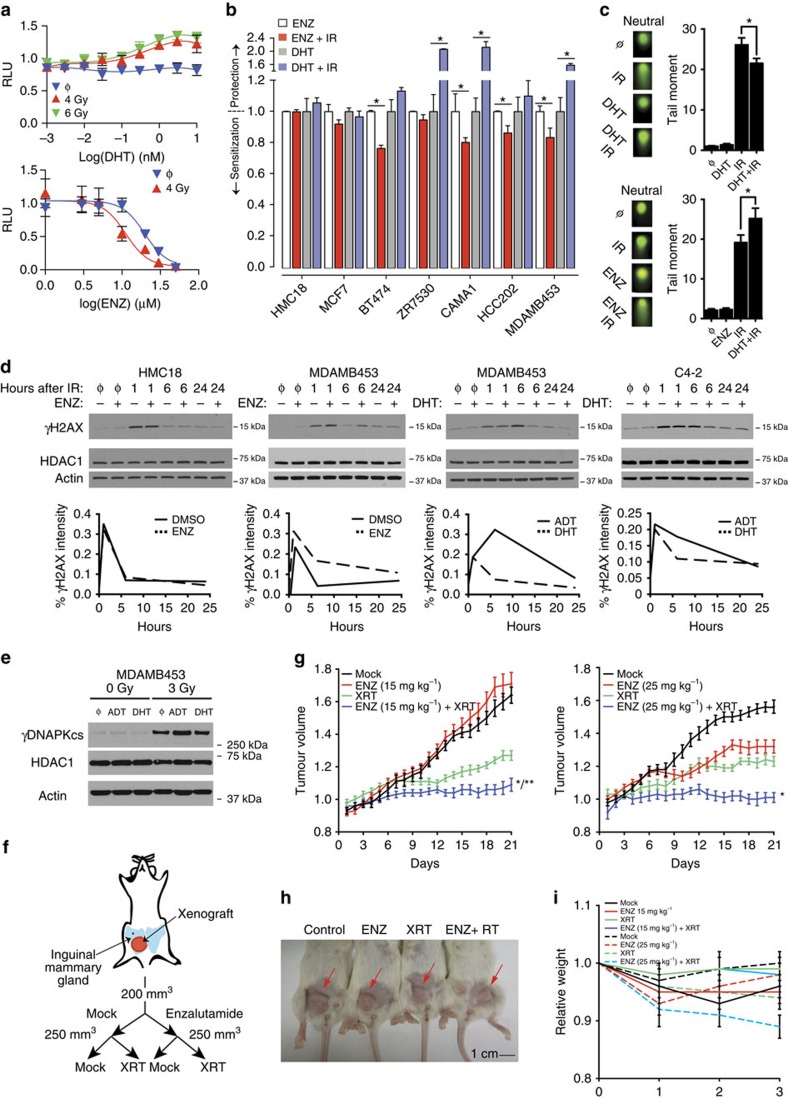
AR activity regulates the response to DNA damage in breast carcinoma. (**a**) (Top) Cells were cultured in steroid-deprived media −/+ DHT for 24 h and then treated with IR: mock (φ), 4 Gy or 6 Gy. Cells were then supplemented with hormone-proficient media at 48 h post-IR. Cell number was determined on day 14–21. (Bottom) Cells were cultured in hormone-proficient media for 24 h without or with enzalutamide (ENZ) and then treated with IR; mock (φ) or 4 Gy. Error bars represent normalized s.e.m. of at least three experiments. (**b**) MCF7, BT474, CAMA1, HCC202 and MDAMB453 cells were treated with ENZ, ENZ+IR, DHT or DHT+IR as in **a**. HMC18 cells were treated similarly but were profiled by clonogenic survival assays. Error bars represent normalized s.e.m. of at least three experiments and the Student's *t*-test was used for statistical analysis. *P<0.05. (**c**) Neutral Comet assay of MDAMB453 cell line, showing increased double-strand breaks when cells were irradiated in steroid-deprived conditions (left) or after 24 h of treatment with 20 μM of ENZ (right). Error bars represent s.e.m. of at least three experiments and the Student's *t*-test was used for statistical analysis. *P<0.05. (**d**) Cells were cultured in hormone-proficient (FBS) media for 24 h −/+ ENZ and then treated with IR: mock (φ), 3 Gy (HMC18 and MDAMB453), or 2 Gy (C4–2). Cells were cultured in steroid-deprived media for 48 h, treated −/+ DHT for 24 h, and then treated with IR: mock (φ), 3 Gy (HMC18 and MDAMB453), or 2 Gy (C4–2). γH2AX, HDAC1, and actin levels were measured at the indicated time points. Relative intensity of γH2AX was calculated by ImageJ64. (**e**) MDAMB453 cells were cultured in steroid-replete, steroid-deficient, or steroid-deficient with 1 nM DHT for 24 h, then treated with irradiation. Cells were harvested and expression of γDNAPKcs was analysed. Androgen deprivation therapy (ADT). (**f**) Schematic of treatment arms. (**g**) MDAMB453 cells were orthotopically injected into the mammary gland of NSG mice and block randomized into one of four treatment arms as shown. Tumour volume was measured daily. Error bars represent normalized s.e.m. of at least seven mice in each treatment arm. *P<0.05 compared with all other treatment conditions based on analysis of variance (ANOVA) and Tukey Contrasts. **P<0.05 for interaction based on two-way ANOVA. (**h**) Pictorial depiction of representative mice from each arm of cohort 2 at the end of treatment. This cohort received ENZ at 25 mg kg^−1^. (**i**) Average weight for each arm in both cohorts was measured weekly. Data are expressed as the means±s.e. of at least seven mice in each treatment arm.

We used MDAMB453 cells to test the interaction between androgen signalling and survival after radiation. Dihydrotestosterone (DHT) re-supplementation of MDAMB453 cells cultured in steroid-deprived media before radiation showed dose dependent rescue of cell growth (Fig. 6a, top). Conversely, ENZ, which competes with DHT for binding to the receptor and prevents its nuclear translocation, decreased cell number compared with vehicle alone (Fig. 6a, bottom). Of note, this experimental system models single fraction radiation treatment. Patients typically receive 16–30 total fractions of treatment, which is predicted to compound the magnitude of the observed interaction between androgen signalling and radiation. We employed the same growth survival assay across several cell lines and showed protection and sensitization with DHT and ENZ, respectively, and only in cell lines that expressed AR and independent of ER and ErbB2 activity (Fig. 6b). For cells capable of forming colonies, these results were confirmed using a clonogenic survival assay (Supplementary Fig. 9).

### *AR* protects breast cancer cells from DNA damage

Using the neutral comet assay, we next determined whether the reduction in cellular survival was associated with increased DNA damage. MDAMB453 cells were pretreated for 24 h with either DHT (Fig. 6c, left) or ENZ (Fig. 6c, right), before 15 Gy of irradiation. These results indicate that DHT decreased and ENZ increased DNA damage in MDAMB453 cells. To assess the role of DNA repair, we measured the kinetics of γH2AX formation and resolution, a surrogate marker of DNA double-strand breaks and subsequent repair, under the same conditions. γH2AX kinetics were consistent with the results of the comet assay: levels of γH2AX in MDAMB453 cells were increased shortly after irradiation by ENZ and decreased by DHT (Fig. 6d), an effect that persisted at 24 h. The kinetics of γH2AX formation and resolution in AR-negative HMC18 cells was unaffected by ENZ treatment, while DHT hastened γH2AX resolution in AR-positive C4–2 prostate cancer cells. These and similar results in the AR-positive, ER-positive, ErbB2 active cell line, BT474 (Supplementary Fig. 10), suggest that suppression of androgen signalling results in increased DNA damage and/or decreased repair in breast cancer cells that express AR, independent of ER and ErbB2 activity.

Previous work on AR regulated radiation resistance in prostate cancer suggested a decrease in the activity of *PRKDC* (or DNAPKcs), a key signalling molecule that initiates early stages of non-homologous end joining, after androgen deprivation^55^. We examined the phosphorylation of DNAPKcs on Ser2056, which is indicative of activated DNAPKcs, in MDAMB453 cells. Cells cultured in steroid-deprived media followed by irradiation had greater γDNAPKcs levels compared with steroid-replete media (Fig. 6e). Supplementation of steroid-deprived media with DHT maintained γDNAPKcs at similar levels to those cells grown in the steroid-replete media following irradiation. The greater level of γDNAPKcs are consistent with increased DNA damage after androgen deprivation in MDAMB453 cells and suggests cell-to-cell or lineage-to-lineage variability in the mechanism by which androgen signalling mediates survival after irradiation. These results demonstrate that androgen activity plays a pivotal role in the response of *AR* expressing breast cancer cells to ionizing radiation.

To assess this concept *in vivo*, MDAMB453 orthotopic xenografts were randomized into one of four treatment groups: mock, ENZ, ionizing radiation, or ENZ and ionizing radiation (Fig. 6f–i). The combination of ENZ (at 15 mg kg^−1^ or 25 mg kg^−1^) and ionizing radiation resulted in more effective suppression of tumour growth than either modality alone. However, despite no effects on tumour growth when used alone, ENZ at a dose 15 mg kg^−1^ showed interactive tumour suppression with radiotherapy. Suppression of tumour growth was observed with ENZ at a dose of 25 mg kg^−1^. However, mice that received both ENZ at a dose of 25 mg kg^−1^ and radiation lost ∼10% of their body weight during the course of treatment, suggesting that selection of ENZ dose is likely to be important in the optimization of the therapeutic index. Taken together, these data show that androgen ablation cooperates with ionizing radiation to decrease tumour cell growth and survival in AR-positive breast adenocarcinomas.

## Discussion

This study represents the largest analysis to date of cancer cell line survival after exposure to ionizing radiation. We profiled 533 genetically annotated tumour cell lines using a single, validated experimental platform and identified genetic determinants of tumour cellular response to radiation. The distributions of survival after exposures to radiation across and within lineages were mostly Gaussian distributions, demonstrating significant underlying biological diversity. The clinical responses of some cancer types to radiotherapy vary in a manner not fully explained by clinical or histopathological features. Our results suggest that intrinsic cellular determinants are likely to contribute to this variance.

We correlated radiation sensitivity and genomic parameters using a statistical methodology that is sensitive to non-linear relationships and with better resolution at the high end of the matching range. We showed that individual SCNA, gene mutations and the basal expression of individual genes and gene sets correlate with radiation survival. In lineage-specific analysis of breast cancer cell lines we demonstrated, for the first time, a role for *AR* expression in the response of breast cancer cells to ionizing radiation, mainly by preventing DNA damage. By studying a large number of cancer types, we found that genetic correlates in any single cancer type can be found in other cancer types as well (for example, Nrf2 activation in LUSC, LUAD and hepatobiliary cancer and *AR* expression in prostate and breast adenocarcinomas). This supports the view that although diverse, cancer genomes reflect combinations of a limited number of functionally relevant events that can confer therapeutic resistance across cancer types. Importantly, the positive correlation between cellular response to ionizing radiation and genotoxic compounds suggests common genetic dependencies between the two most common cancer therapies in use, DNA-damaging chemotherapy and X-rays.

We identified several new genetic determinants of response to DNA damage. These genetic alterations can have predictive capacity by identifying the likelihood of response to therapy and, consequently, prognosis. Diagnostics that measure genetic changes can assist in the selection of patients likely to respond to anti-cancer agents^4^,^5^,^6^,^7^,^56^. The potential for stratification of patients from heterogeneous populations to genetically similar subgroups can help guide the transition of DNA-damaging chemotherapy and X-rays from a generic population-based approach to one that is more personalized. A subset of the alterations that we identified can also guide combinatorial therapeutic strategies since these alterations conferred resistance and are targets of current FDA approved drugs, creating an opportunity for the precision targeting of therapeutic resistance (for example, AR expression and anti-androgens in breast cancer and *NFE2L2* activation and anti-PI3K therapies in NSCLC^8^,^57^,^58^).

Although we identified several genetic determinants that regulate the survival of cells after exposure to radiation, there are surely substantial additional parameters. Many of the cancer types we profiled were represented by relatively few samples and others were not represented. Some SCNA were not measured due to the resolution limit of the array platform and although the input of >1,600 cancer relevant genes profiled for mutations provides a powerful initial assessment of likely relevant genes, it is not comprehensive. Finally, levels of non-coding RNA, metabolites and proteins and their post-translational modifications are also likely to impact the intrinsic cellular response to radiation. Our data enables future correlations of ionizing radiation sensitivity with levels of these important biomolecules as additional genomic and cellular datasets emerge.

In summary, our results reveal a genetic basis of the variation in the vulnerability of cancer to DNA damage. This information can help guide the transition of the use of X-rays and DNA-damaging drugs from the current generic approach to one in which these therapies are guided by genetic alterations in individual patient's tumour.

## Methods

### Cell line validation

Cell lines from the Broad Biological Samples Platform were thawed and tested for survival after irradiation between January 2012 and February 2013. Cells were grown in media (Supplementary Data 1) supplemented with 10% fetal bovine serum (Thermo Fisher, MA) and 100 U ml^−1^ Penicillin, 100 μg ml^−1^ of Streptomycin, and 292 μg ml^−1^ L-glutamine (Corning, NY). When a reference single-nucleotide polymorphism (SNP) genotype was available for a cancer cell line through the CCLE project, we conducted SNP genotyping by Fluidigm^59^. At the time of publication, we have positively matched 87.8% of the 533 cancer cell lines analysed in this study to their reference genotype (Supplementary Data 1). As additional samples are matched, we will provide updated information and analyses reflecting any changes at the CTD^2^ Data Portal (ctd2.nci.nih.gov/dataPortal/) and in CTRP v2. For cellular validation studies, C4–2 cells were from the laboratory of Karen E. Knudsen (Thomas Jefferson University) and HEC59 cells were from the laboratory of Thomas Kunkel (NIES). We cross-referenced our cell lines with the database of cross-contaminated or misidentified cell lines curated by the International Cell Line Authentication Committee and NCBI BioSample and identified six cell lines that could have been contaminated or misidentified: BT20, J82, JHH1, MDAMB435S, MKN7 and RT4. All six of these cell lines were SNP gentoyped and confirmed to match the references genotype (Supplementary Data 1).

### Cell culture and irradiation

All cultures were maintained at 37 °C in a humidified 5% CO_2_ atmosphere and tested to ensure absence of *Mycoplasma*. Plates were treated with γ-radiation delivered at 0.91 Gy min^−1^ with a ^137^Cs source using a GammaCell 40 Exactor (Best Theratronics; Ontario, Canada).

### Antibody and reagents

Anti-AKT (clone C67E7, #4691P, 1:1,000), anti-phospo-S473-AKT (clone D9E, #4060P, 1:1,000), anti-AR (clone D6F11, #5153, 1:2,000), anti-HDAC1 (clone 10E2, #5356, 1:2,000), anti-γH2AX (clone 20E3, #9718, 1:2,000), anti-actin (clone 8H10D10, #3700, 1:4,000), anti-γErbB2 (clone Tyr1248, #2247, 1:1,000), and anti-GAPDH (clone D16H11, #5174, 1:4,000–7,500) were from Cell Signalling Technology (Beverly, MA). Anti-HER2 (clone e2–4001, #MS730P0, 1:2,000) and anti-ER (clone AB-17, #RB1521PO, 1:1,500) were from Thermo Fisher (Waltham, MA). Anti-γDNP-PKcs (clone S2056, #18192, 1:1,000 was from Abcam (Cambridge, MA). Enzalutamide was from Selleck (Houston, TX). DHT was from Steraloids (Newport, RI).

### High-throughput proliferation assay

Cells were plated using a Multidrop Combi liquid handler (Thermo Fisher) in at least quadruplicates for each time point at three cell densities (range 25–225 cells per well) in a white 384-well plate (Corning, NY). Plates were irradiated and at 9 days post irradiation, media was aspirated and 40 μl of CellTiter-Glo reagent (50% solution in PBS; Promega, WI) was added to each well. Relative luminescence units were measured using an Envision multilabel plate reader (Perkin Elmer) with a measurement time of 0.1 s. Luminescence signal is proportional to the amount of ATP present. For chemical radiosensitization measurements, drug was added 24 h before irradiation. The luminescence signal was plotted as a function of cell density and a cell density within the linear range for luminescence (or growth) was selected to generate integral survival for each cell line (see also Supplementary Data in ref. 8).

### Integral survival

The area under the curve was estimated by trapezoidal approximation. First, *x* axis values representing radiation doses 1, 2, 3, 4, 5, 6, 8, and 10 Gy were log_2_ transformed. The survival values for each trapezoid were multiplied by the dose interval, [*f*(*X*_1_)+*f*(*X*_2_)/2] × Δ*X*, summed and re-scaled by multiplying by (7÷log_2_10) so that integral survival is defined from 0 (completely sensitive) to 7 (completely resistant).

### Clonogenic survival

Cells were plated at appropriate dilutions, irradiated and incubated for 7–21 days for colony formation. For chemical radiosensitization measurements, drug was added 24 h before irradiation. Colonies were fixed in a solution of acetic acid and methanol 1:3 (v/v) and stained with 0.5% (w/v) crystal violet as previously described^60^. A colony was defined to consist of 50 cells or greater. Colonies were counted digitally using ImageJ software as described^61^. Integration of survival as a function of dose, or area under the curve, was calculated using Prism, GraphPad Software (La Jolla, CA).

### Information-based association score

The association between genomic alterations (for example, mutations or SCNA) or ssGSEA profiles for each gene set and the radiation response profile was determined using the IC^8^,^62^,^63^.

### Genetic data

Cancer cell lines were profiled at the genomic level and the processed data are available for download at http://www.broadinstitute.org/ccle (ref. 64). Briefly, mutation information was obtained both by using massively parallel sequencing of >1,600 genes and by mass spectrometric genotyping (OncoMap 3.0), which interrogated 381 specific mutations in 33 known oncogenes and tumour suppressors. Genotypes were transformed to categorical values (mutation=1, no mutation=0) and were used as input to compute the IC.

Genotyping/copy number analysis was performed using Affymetrix Genome-Wide Human SNP Array 6.0. Raw Affymetrix CEL files were converted to a single value for each probe set representing a SNP allele or a copy number probe using a GenePattern pipeline^65^ and hg18 Affymetrix probe annotations. Copy numbers were then inferred based on estimating probe set-specific linear calibration curves, followed by normalization by the most similar HapMap normal samples. Segmentation of normalized log_2_ ratios (specifically, log_2_(CN/2)) was performed using the circular binary segmentation algorithm^66^, followed by median centring of the segment values to a value of zero in each sample. Next, quality checking of each array was performed, including visual inspection of the array pseudo-images, probe-to-probe noise variation between copy number values, confidence levels of Birdseed^67^ genotyping calls, and appropriate segmentation of the copy number profiles. Finally, the GISTIC algorithm^68^ was used to identify focal regions of CNAs in individual samples. A gene-level copy number was also generated, defined as the maximum absolute-segmented value between the gene's genomic coordinates, and calculated for all genes using the hg18 coordinates provided by the refFlat and wgRna databases from UCSC Genome Browser (http://hgdownload.cse.ucsc.edu/goldenPath/hg18/database/). Separate binary variables representing amplifications (above 0.7) and deletions (below −0.7) were generated based on the GISTIC gene-level copy number output described above. These binary amplification/deletion variables for each gene were used as input to compute the IC against the radiation sensitivity phenotype.

mRNA gene expression was measured by the GeneChip Human Genome U133 Plus 2.0 Array. Raw Affymetrix CEL files were converted to a single value for each probe set using Robust Multi-array Average (RMA) and normalized using quantile normalization. Either the original Affymetrix U133+2 CDF file or a redefined custom CDF (http://brainarray.mbni.med.umich.edu/Brainarray/Database/CustomCDF/CDF_download.asp) file (ENTREZG—v15) was used for the summarization. ssGSEA enrichment scores were calculated based on the weighted difference of the Empirical Cumulative Distribution Functions of the genes in the set relative to the genes not included in an individual set^33^. The result is a single score per cell line per gene set, transforming the original dataset into a more interpretable higher-level description. Gene sets were obtained from the C2 sub-collection of the Molecular Signatures database (MSigDB)^69^, an additional collection of oncogenic signatures, and other cancer-related gene sets curated from the literature, resulting in a dataset that has 4,628 pathway profiles for each sample. ssGSEA values were used as input to compute the IC.

The nominal *P* values for the information-based association metric scores between the genetic parameters (alterations or ssGSEA scores) and radiation response scores were estimated using an empirical permutation test.

### *NFE2L2* pathway signatures

For the gene transcription signature of pathway *NFE2L2* (or NRF2), we extracted the expression values from the CCLE dataset. For each gene, we normalized expression values to standard deviations from the median across cell lines. We computed the average normalized expression of the signature genes within each cell line in which data was available. Across the cell lines, we normalized the gene signature scores to standard deviations from the median across CCLE, and a ‘summary score' for each pathway was computed as the average of the individual normalized signature scores^70^.

### Comet assays

Single-cell gel electrophoresis was conducted in alkaline or neutral buffer according to the manufacturer's instructions; Trevigen (Gaithersburg, MD). Slides were blinded and enumerated by a single user.

### Western blot analysis

Whole-cell lysates were prepared using M-PER lysis buffer and clarified by centrifugation. Proteins were separated by SDS–PAGE and transferred onto 0.45 μM nitrocellulose membranes (Maine Manufacturing; Sanford, ME). After primary antibody incubation for 1–2 h at room temperature, washings, and incubation with secondary antibodies, blots were developed with a chemoluminescence system (Amersham/GE Healthcare). For γH2AX measurements, proteins were transferred onto 0.2 μM nitrocellulose (Bio-Rad). Cropped blots are displayed in the main figures and some full-length blots are presented in Supplementary Fig. 11.

### Mouse xenograft studies

Female NSG mice, 6–8 weeks of age, were obtained from the Cleveland Clinic Biological Resources Unit facility. All mouse studies were conducted under a protocol approved by the Cleveland Clinic Institutional Animal Care and Use Committee. MDAMB453 cells were resuspended in serum free media and injected into the inguinal mammary gland. Once tumours reached 200 mm^3^, mice were block randomized and assigned to vehicle, ENZ, vehicle plus radiotherapy, or ENZ plus radiotherapy. Two cohorts consisting of these four arms underwent treatment. Vehicle consisted of a volume of 5 ml per kg of PEG-400 solution containing 1.5% of dimethylsulphoxide for cohort 1 and 2.5% dimethylsulphoxide for cohort 2 via oral gavage daily. Cohort 1 received ENZ at 15 mg kg^−1^ and cohort 2 received enzaluatmide at 25 mg kg^−1^. Radiotherapy was delivered to a dose of 1.5 Gy in three fractions once tumour size reached 250 mm^3^. Treatment was not blinded to the investigator. Tumour volume was measured daily. Mice were sacrificed once their tumours reached an approximate size of 1,000 mm^3^ or at treatment days 21–28. The significance of the difference between treatment groups was assessed by one-way and the interaction between drug and radiation was measured by two-way analysis of variance.

## Additional information

**How to cite this article:** Yard, B. D. *et al*. A genetic basis for the variation in the vulnerability of cancer to DNA damage. *Nat. Commun.* 7:11428 doi: 10.1038/ncomms11428 (2016).

## Supplementary Material

Supplementary Information Supplementary Figures 1-11.

Supplementary Data 1 CCLE cell lines profiled and radiation sensitivity data

Supplementary Data 2 Pearson and Spearman correlation coefficients for the comparison of X-rays and drugs.

Supplementary Data 3 fSCNA, mutation number, and radiation sensitivity data.

Supplementary Data 4 SCNA and survival after irradiation.

Supplementary Data 5 Gene mutations and survival after irradiation.

Supplementary Data 6 ssGSEA and survival after irradiation.

Supplementary Data 7 Correlation between indiviudal gene expression and survival after irradiation.

Supplementary Data 8 SCNA, gene mutations, and survival after irradiation in breast carcinoma.

Supplementary Data 9 Selected SCNA and gene expression values and survival after irradiation in breast carcinoma.

Supplementary Data 10 ssGSEA and survival after irradiation in breast carcinoma (top 27 gene sets).
