# Supplementary material for: A genetic basis for the variation in the vulnerability of cancer to DNA damage
Source: Nat Commun. 2016 Apr 25;7:11428. doi: 10.1038/ncomms11428 (PMC4848553; doi:10.1038/ncomms11428)
Supplement: Supplementary Information — Supplementary Figures 1-11. [file ncomms11428-s1.pdf]

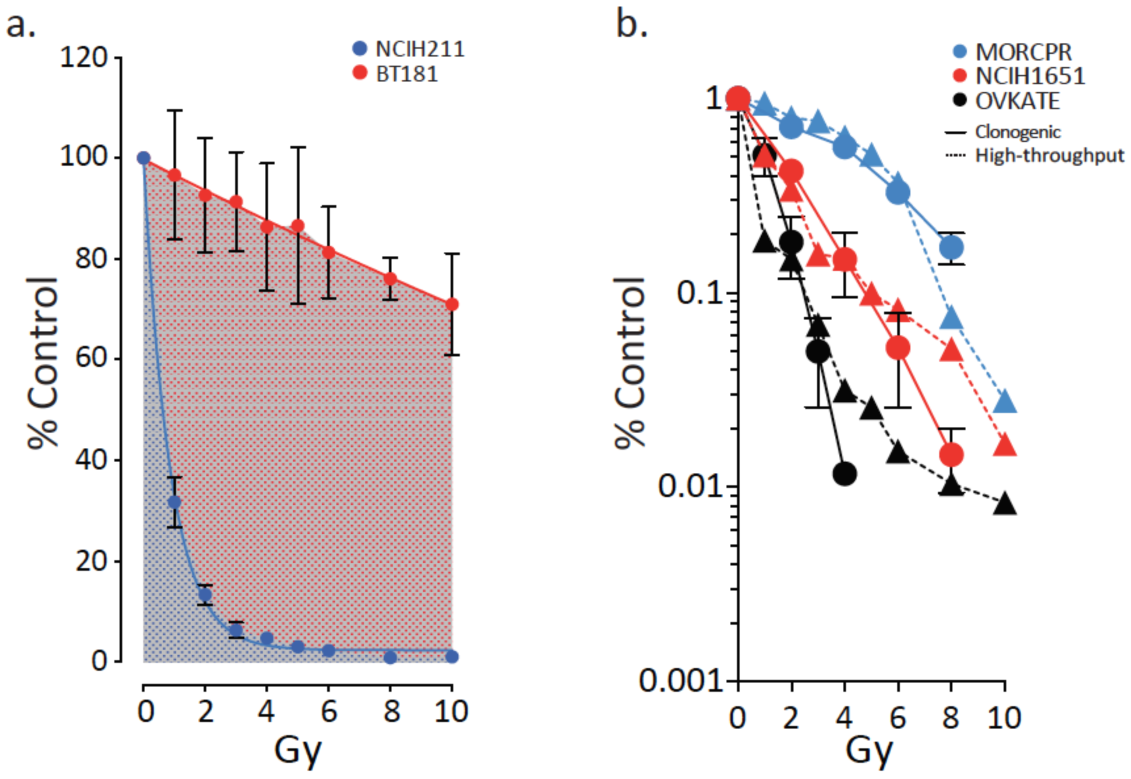

Supplementary Figure 1: High-throughput profiling of survival after exposure to γ-radiation. (a) Cells were plated in at least 7 wells in a 384-well plate at cell densities ranging from 25-225 cells in 70 μL of media per well for 24 hours and then treated with IR: 0 (mock), 1, 2, 3, 4, 5, 6, 8, or 10 Gy. Luminescence-based detection of cellular ATP, a surrogate measure of cell number, was performed after 9 days after radiation treatment. Proliferation fraction as a function of dose for NCIH211 and BT181 after exposure to γ-rays is shown. Integral survival (shaded area) was estimated by the trapezoidal approximation of area under the curve (see *Methods*). Data are expressed as the means ± s.d of at least four replicates. (b) Correlation between high-throughput platform ($n = 1$) and clonogenic survival measurements ($n \geq 2$) across individual doses. The proliferating fraction (single-run) and clonogenic survival (for each cell line, $n \geq 2$) were plotted as function of dose. The proliferation index closely approximated clonogenic survival, especially within the GI_{50} range. High-throughput platform data are expressed as the means of at least four replicates. Clonogenic survival data are expressed as the means ± s.e.m of at least three experiments.

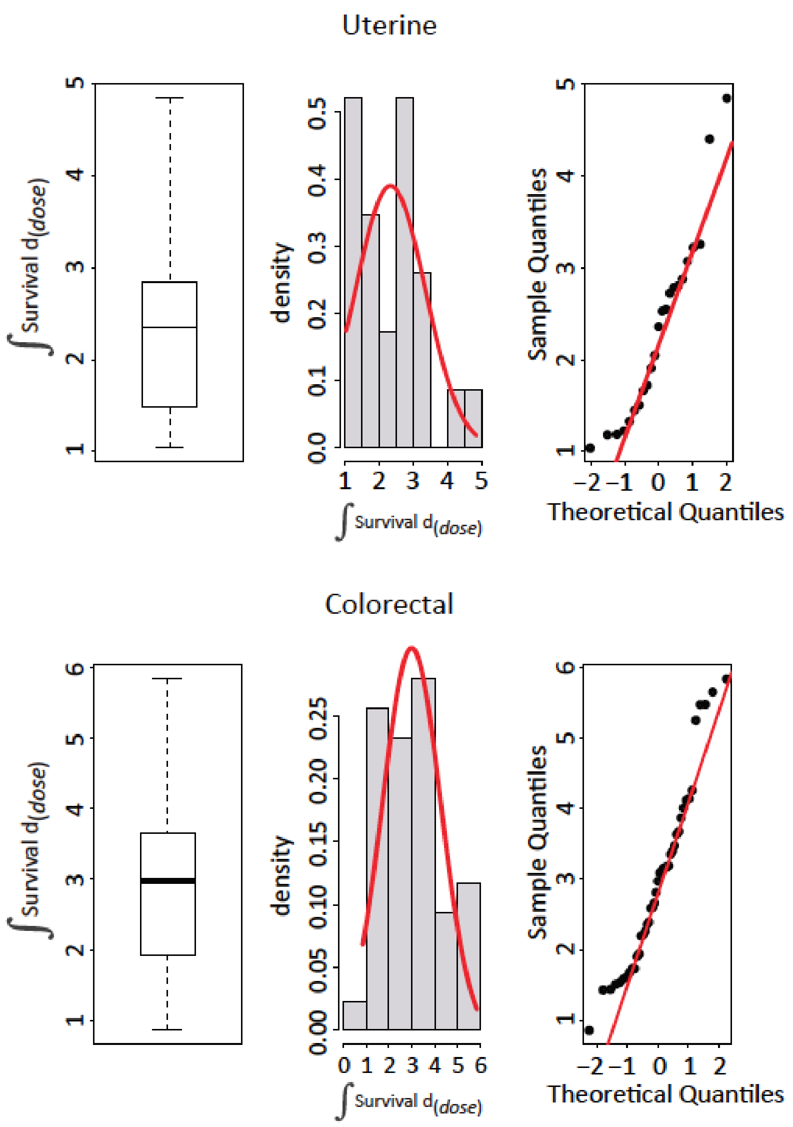

Supplementary Figure 2: Non-*Gaussian* variation in uterine and colorectal carcinoma cell line survival after radiation-induced damage. Box plot and whiskers plots, histogram, probability density function, and Normal Q-Q plots analyses of calculated integral survival for cell lines derived from the uterine and colorectal carcinomas. Whiskers represent minimum and maximum integral survival values in each distribution. The horizontal line in the boxplot represents the median. Histogram analysis of the uterine lineage suggests a bimodal distribution.

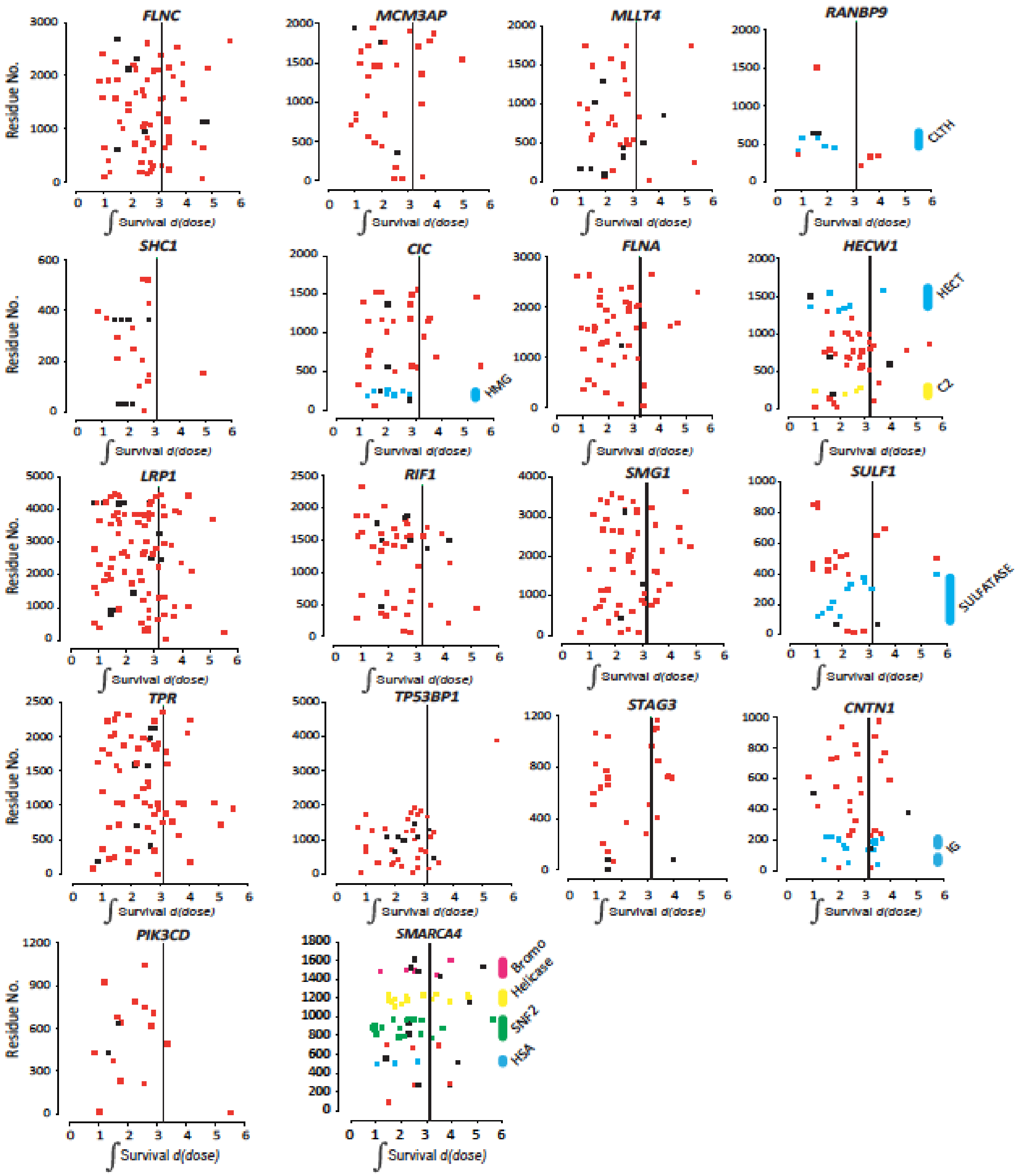

Supplementary Figure 3: Mutations in a subset of genes associated with radiation sensitivity demonstrate domain-selectivity in conferring sensitivity. Scatter plot of integral survival and amino acid position for cell lines containing mutations in the designated genes. Protein domains predicted by InterPro (http://www.ebi.ac.uk/interpro/) and/or ExPasy (http://prosite.expasy.org/) are shown.

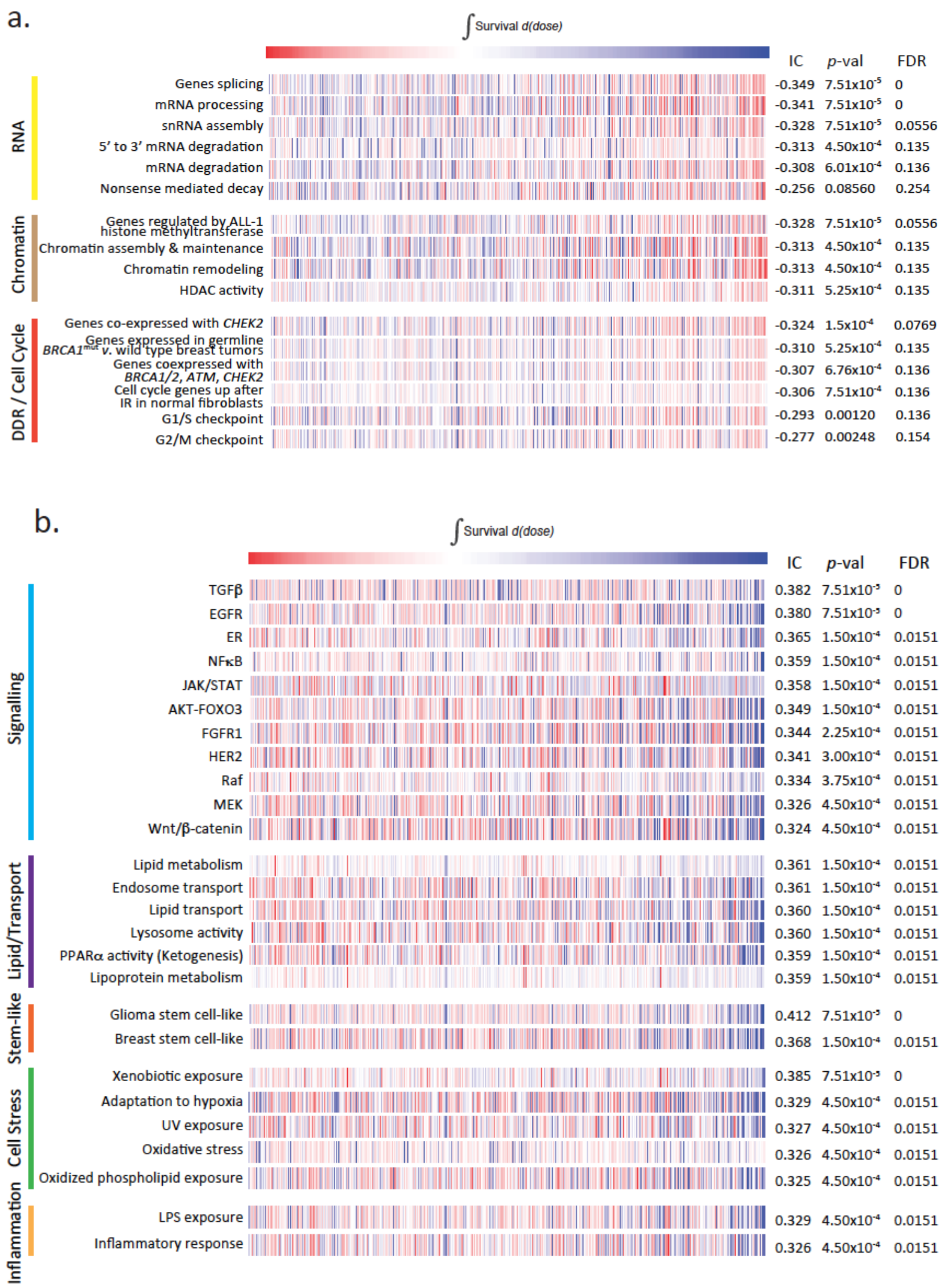

Supplementary Figure 4: ssGSEA identifies gene sets that correlate with radiation sensitivity (a) and resistance (b). Heat map of ssGSEA scores (red = positive, blue = negative). Top gene sets, organized by biological processes, are shown.

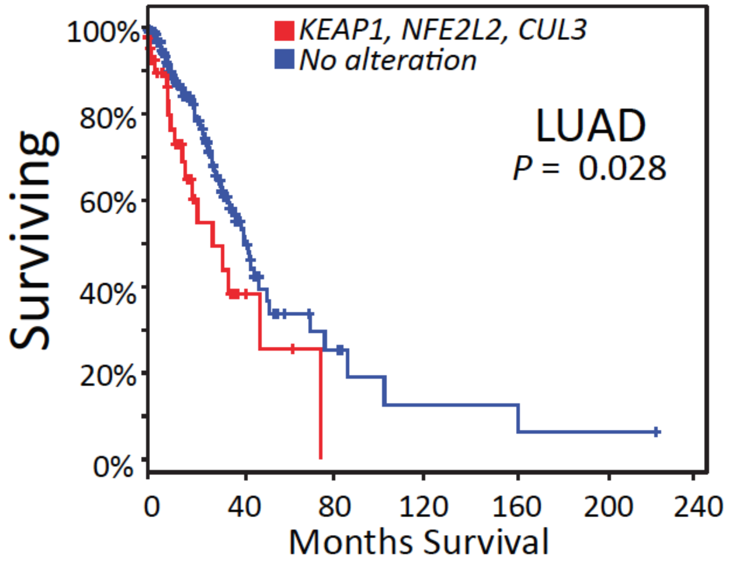

	total cases	deceased	median survival
# with alteration	46	22	32.70
# without alteration	157	41	45.31

Supplementary Figure 5: Kaplan-Meier survival analysis curve calculated from lung adenocarcinoma patients from TCGA (https://tcga-data.nci.nih.gov/tcga/) separated by *NFE2L2* (mutation or CNA), *KEAP1* (mutation or CNA), and *CUL3* homozygous deletion versus none of these alterations. There was a statistically significant difference in overall survival by log-rank test (P = 0.028).

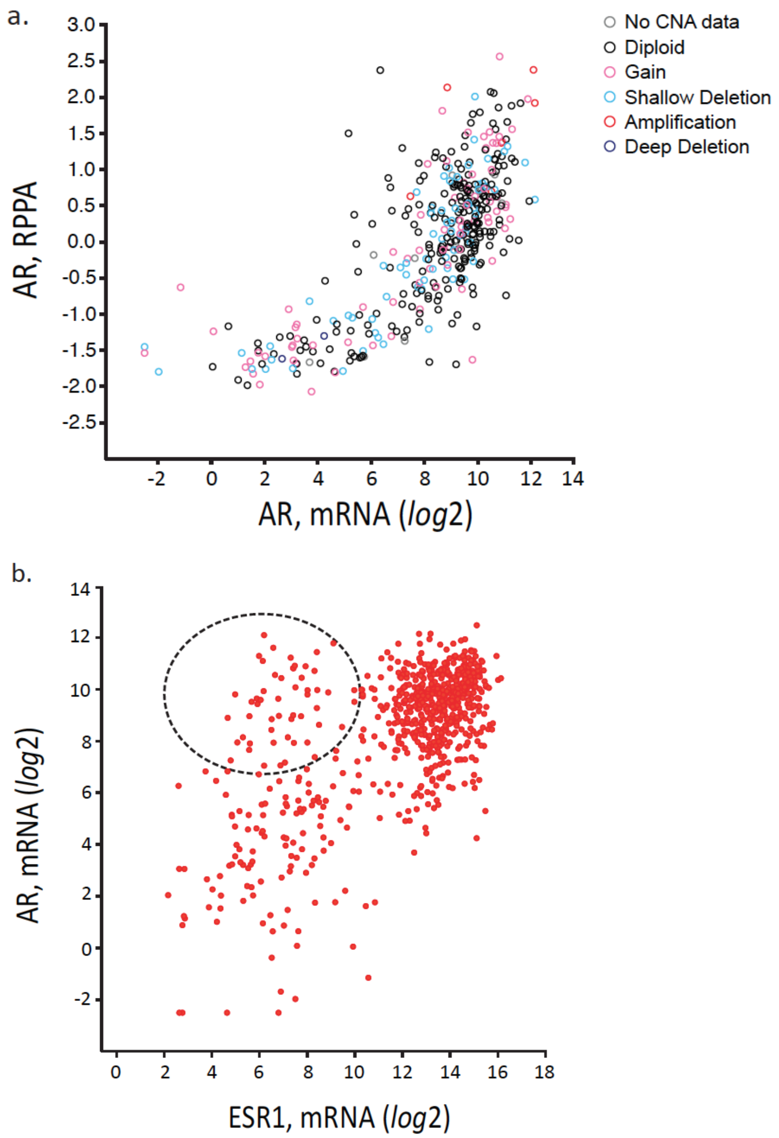

Supplementary Figure 6: Correlation of *AR* and *ESR1* expression. (a) AR protein levels correlate with mRNA levels (Pearson r = 0.615). AR overexpression is mostly observed in diploid tumor samples, suggesting that increased AR expression is mainly regulated by increased gene transcription rather than gene amplification. AR protein was measured by reverse phase protein array (RPPA) and mRNA measured by RNA-Seq from 1098 breast cancer patient samples profiled by TCGA (https://tcga-data.nci.nih.gov/tcga/) and analyzed by (cbioportal.org). (b) Scatter plot and regression of AR and ESR1 mRNA expression are shown (Spearman r = 0.51). Breast tumors that express AR and not ESR1 were determined using a cut-off of the median for AR and ESR1 expression and delimited by a dashed circle. RNA-Seq expression values were calculated by expectation maximization (RSEM) and scaled by *log*2 transformation.

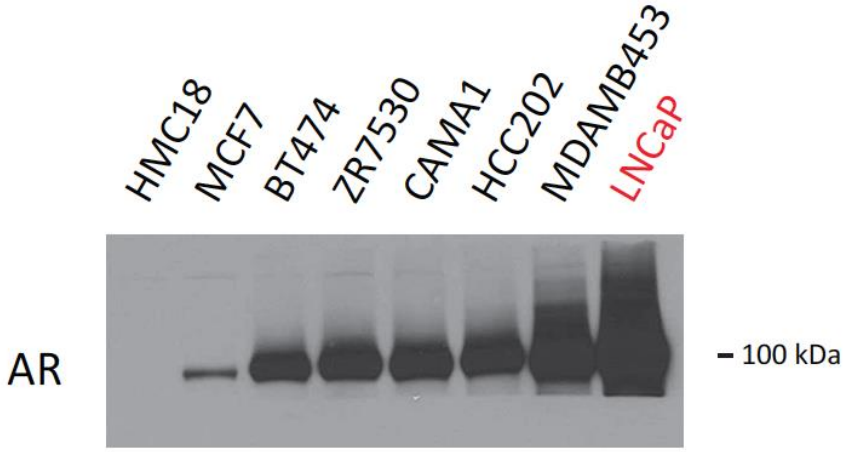

Supplementary Figure 7: Relative AR protein levels in whole cell extracts from breast cancer cell lines and LNCaP, a prostate cancer cell line. Increased time of film exposure reveals that MCF7 cells express low levels of AR (compare to Fig. 5f). HMC18 cells do not express AR protein.

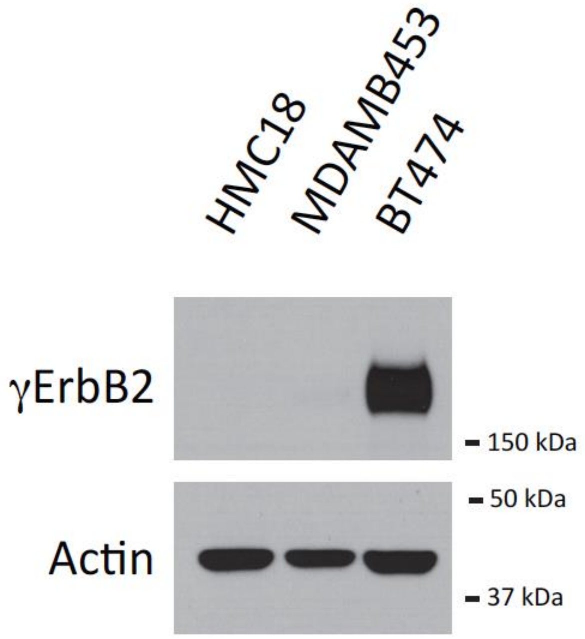

Supplementary Figure 8: ErbB2 is overexpressed but not activated in MDAMB453 cells. γErbB2 and actin levels were measured in whole cell extracts from HMC18, MDAMB453, and BT474 cells.

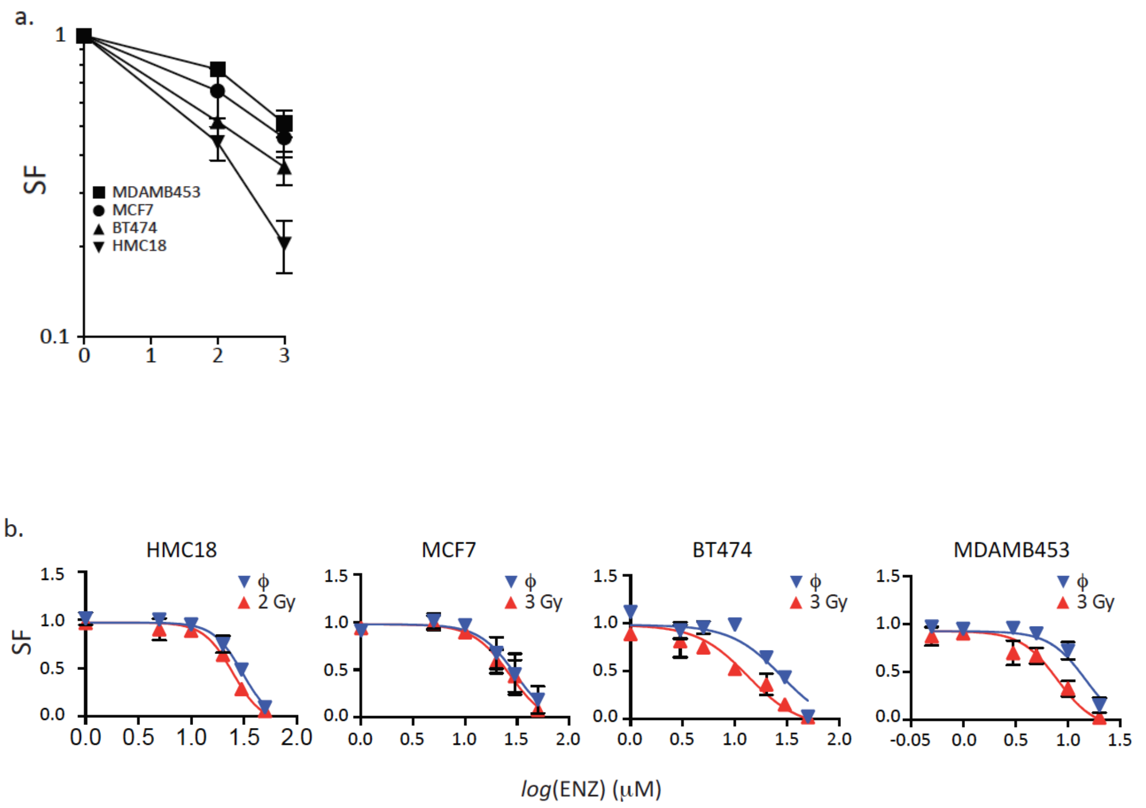

Supplementary Figure 9: Clonogenic survival measurements in breast cancer cells. (a) Clonogenic survival measurements of breast cancer cells HMC18, MCF7, BT474, and MDAMB453. Colony number was determined on days 7-21. Data are expressed as the means ± s.e.m of at least three experiments. (b) Clonogenic survival measurement of enzalutamide-mediated radiosensitization in cell lines HMC18, MCF7, BT474, and MDAMB453. Cells were cultured in hormone-proficient (FBS) media for 24 hours without or with enzalutamide (ENZ) and then treated with IR; mock (ϕ) or radiation. Colony number was determined on days 7-21. Data are expressed as the means ± s.e.m of at least three experiments.

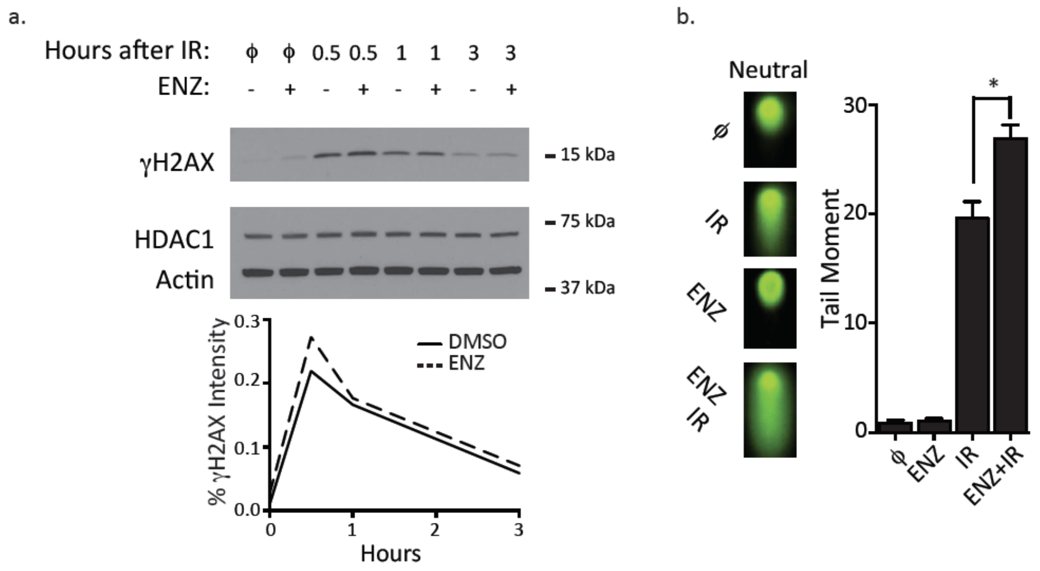

Supplementary Figure 10: AR regulates DNA damage and repair in AR, ER positive cells. (a) BT474 cells were cultured in hormone-proficient (FBS) media for 24 hours -/+ ENZ (20 μM) and then treated with IR: mock (ϕ) or 4 Gy. γH2AX, HDAC1, and Actin levels were measured at the indicated time points. Relative intensity of γH2AX was calculated by ImageJ64 as described (ref). (b) Neutral Comet assay of BT474 cells, showing increased double-strand breaks when cells were irradiated (10 Gy) after 24 hours of treatment with ENZ (20 μM). Data are expressed as the means ± s.e.m of at least three experiments.

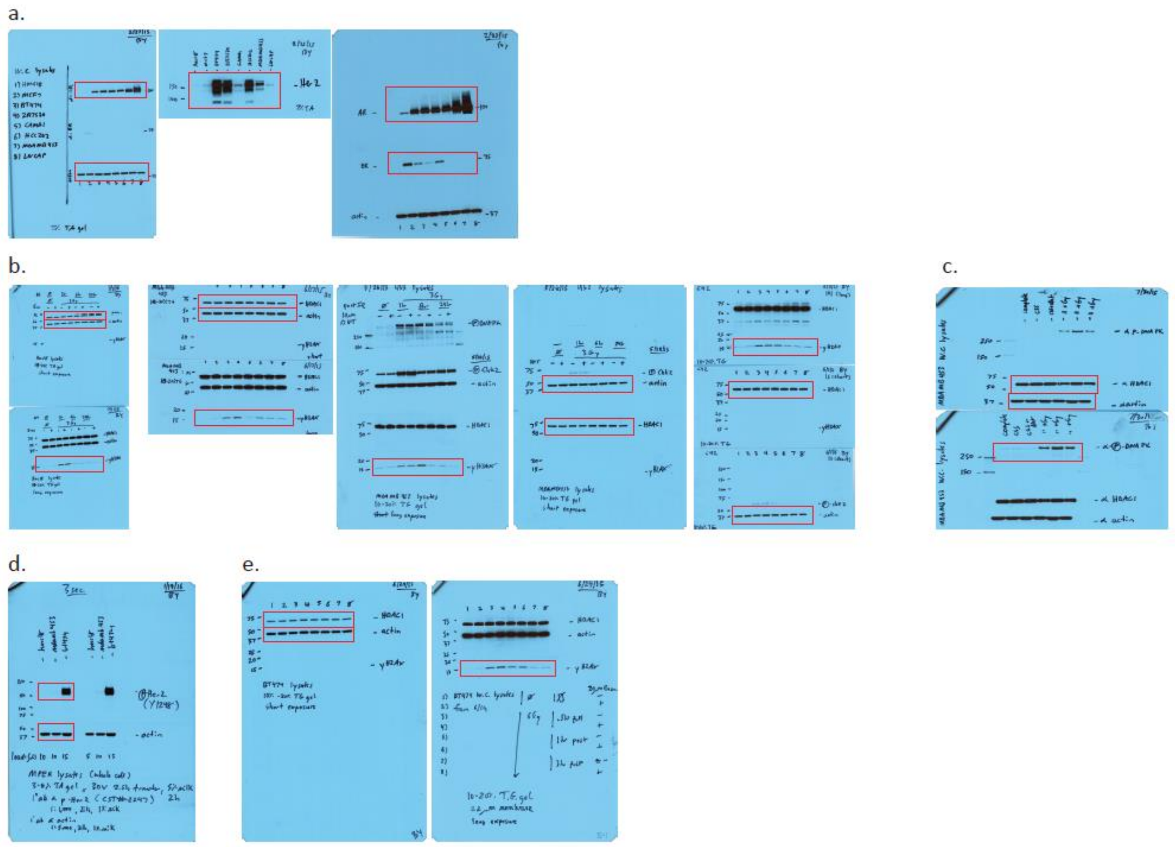

Supplementary Figure 11: Full images of cropped blots presented in other figures. (a) Full blot for Figure 5f and Supplementary Figure 7. Over-exposition of the blot was necessary to reveal the low AR signal in MCF-7. (b) Full blot for Figure 6d. (c) Full blot for Figure 6e. (d) Full blot for Supplementary Figure 8. (e) Full blot for Supplementary Figure 10.
